# Effects of Different Probiotics on Laying Performance, Egg Quality, Oxidative Status, and Gut Health in Laying Hens

**DOI:** 10.3390/ani9121110

**Published:** 2019-12-10

**Authors:** Quanhang Xiang, Chao Wang, Hong Zhang, Wen Lai, Hongkui Wei, Jian Peng

**Affiliations:** 1 Department of Animal Nutrition and Feed Science, College of Animal Science and Technology, Huazhong Agricultural University, Wuhan 430070, China; xiangquanhang@webmail.hzau.edu.cn (Q.X.); wangchao1028@163.com (C.W.); laiwen@zhengbang.com (W.L.); weihongkui@mail.hzau.edu.cn (H.W.); 2 The Cooperative Innovation Center for Sustainable Pig Production, Wuhan 430070, Hubei, China

**Keywords:** *C. butyricum*, *S. boulardii*, *P. acidilactici*, laying performance, egg quality, gut health, laying hens

## Abstract

**Simple Summary:**

Resistance has developed against almost all the main classes of antibiotics, and finding efficient alternatives to these antibiotics is urgently required. Based on previous research, three types of probiotic bacteria were chosen to be administrated in a laying hen diet. We found that the performance, egg quality, and gut health of laying hens were improved after probiotic treatment.

**Abstract:**

With recent bans on the growth-promoting use of antibiotics, alternative strategies are needed to improve the performance of agricultural animals. Here, the effects of dietary supplementation with *Clostridium butyricum* and a combination of *Saccharomyces boulardii* and *Pediococcus acidilactici* were assessed on laying performance, egg quality, oxidative status, and gut health in laying hens. A total of 8208 Lohmann pink laying hens were divided into 3 treatment groups, with each group replicated 12 times (*n* = 228). Hens in the control group (CON) were provided a basic diet devoid of added antibiotics and probiotics. Treatment group 1 (T1) received the same base diet supplemented with 0.5 g/kg *C. butyricum*, and the diets of treatment group 2 (T2) supplemented with *S. boulardii* (0.05 g/kg) and *P. acidilactici* (0.1 g/kg) for the entirety of the 5-week trial. The data indicated that *C. butyricum* supplementation resulted in a significant reduction in ADFI, a significant increase in feed conversion, eggshell strength, and the CP% of albumen (dry matter, DM) relative to CON. The probiotic-treated hens exhibited decreased reactive oxygen species (ROS) levels in ileum and cecum, and reduced malondialdehyde (MDA) in serum. In conclusion, dietary supplementation with *C. butyricum* may be beneficial with respect to hen performance, egg quality, and gut health.

## 1. Introduction

Antibiotics have been recognized as one of the most effective therapies in medicine [1,2]. However, due to the growing number of antibiotic-resistant bacteria, the agricultural use of antibiotics as growth promoters in livestock has being banned in many countries worldwide. Antibiotic growth promoters (AGPs) have been banned in the European Union since 2006 [3]. In spite of the prohibition, a large number of AGPs are being administered to laying hens, through individual treatments or water and diet supplementation, in an effort to reduce the occurrence of diseases and improve the performance of laying hens [4,5]. Not only can overuse of antibiotics at subtherapeutic doses lead to bacterial resistance [6,7,8], but they are also detectable in eggs. This poses significant concerns regarding the potential effects on human health [9]. Therefore, alternatives to antibiotics are urgently needed.

It has been documented that probiotics are an attractive alternative to antibiotics which have been demonstrated to improve intestinal health, increase the stability of the gut flora, and suppress the colonization of pathogens [10,11]. Previous reports have indicated that dietary supplementation with probiotics is not only capable of increasing egg production, but also improving feed conversion efficiency [12], promoting hen performance, and eggshell quality [13]. Moreover, it was reported that probiotics could also regulate symbiotic bacteria colonization [14], increase the number of intestinal goblet cells [15], and stimulate intestinal T-cell immunity [16]. These findings provide intriguing evidence that probiotics may have a beneficial effect on laying hens. *Clostridium butyricum* is an anaerobic bacillus, which has been approved as a feed additive for broilers and weaned piglets in the European Union since 2003. In addition, *C. butyricum* was also approved as a feed additive for laying hens. In mouse studies, *C. butyricum* supplementation increased the numbers of T_reg_ cells in the intestinal lamina propria, promoted the secretion of the key anti-inflammatory cytokine IL-10, and improved the early development of intestinal immune tolerance [17]. Of particular interest is a study by Zhang et al. (2014), in which the authors demonstrated that *C. butyricum* supplementation was able to improve the growth performance, immune function, and cecal microflora in broiler chickens following treatment with *E. coli* K88 [18]. However, studies examining the effects of *C. butyricum* on laying hens are limited.

Previous studies have demonstrated that dietary yeast products could improve intestinal immunity [19,20,21] and minimize coccidial infection in layer chickens [22]. *S. boulardii,* a relative of *Saccharomyces cerevisiae*, is a recognized probiotic fungus. As a facultative anaerobic fungus, *S. boulardii* can provide nutrients for the host, improve the activity of beneficial intestinal bacteria, inhibit the growth of pathogens, and improve the immune function of the intestinal mucosa [23,24]. *P. acidilactici* was reported to produce specific lactic acid products, which was widely used in improving productive performance of laying hens [13,25]. As *P. acidilactici* is a strict anaerobic probiotic, *S. boulardii* may improve the function of *P. acidilactici* by accelerating intestinal oxygen consumption.

The objectives of this study were to (1) evaluate the effects of *C. butyricum* and a combination of *S. boulardii* and *P. acidilactici* on the performance, egg quality, morbidity, and mortality of laying hens, and (2) to study the effect of probiotics on the gut microbiota and the intestinal structure and function of laying hens.

## 2. Materials and Methods

### 2.1. Probiotic Strains

Three pure probiotic strains were used in this study. *C. butyricum* was provided by Hubei Lvxue Biotechnology Co., Ltd. (Xianning, Hubei, China) Powdered *C. butyricum* is provided. *S. boulardii* I-1079 and *P. acidilactici* MA18/5M were provided by Beijing Hilink International Biotechnology Co., Ltd. (Beijing, China) The dose of dietary probiotic supplement was added with reference to the companies’ commercial recommendations.

### 2.2. Experimental Design

The protocol for the animal experimental procedures was approved by Institutional Animal Care and Use Committee of Huazhong Agricultural University (Wuhan, China). The ethical number of this study is HZAUCH-2017-009.

A total of 8208 Lohmann pink laying hens (180 days) were randomly assigned to 3 groups with 12 replicates of 228 birds each (2736 laying hens per group). The 3 groups were divided into one control group (CON) and 2 treatment groups (T1 and T2). The diets for T1 consisted of a standard feed supplemented with *C. butyricum* (0.5 g/kg), and that of T2 was supplemented with both *S. boulardii* (0.05 g/kg) and *P. acidilactici* (0.1 g/kg).

### 2.3. Birds, Diet and Management

This trial was carried out in Xian Ning, Hubei Province, during the month of January. Three birds were housed in individual cages, under a 16 h light/8 h dark cycle (lights on at 5:00, lights off at 21:00) and a constant temperature of 9 ± 2 °C. All birds were acclimated to a basal diet for 1 week. laying hens were caged in 3-tier battery cages, 3 hens per cage. The basal diets provided to the hens are presented in Table 1. The temperature, humidity, and light conditions in different chicken coops were similar. Water and feed were provided ad libitum during the 5-week study period.

### 2.4. Sample Collection and Analytical Determination

#### 2.4.1. Samples

Health condition, mortality, feed intake, laying performance, and egg quality were assessed daily. At the end of the trial, a single healthy laying hen was selected from each group replicate, 12 in each group, for a total of 48 hens, which were then sacrificed. Blood and intestinal samples were collected for examination. Blood samples were collected from the axillary vein into vacuum tubes (10 mL) containing coagulant. The blood was then centrifuged at 3000× *g* for 10 min at 4 °C. Serum was collected and stored at −80 °C until the time of further analysis. The gut of each hen was removed immediately after sacrifice, and segments of ileum and cecum were identified and ligated prior to excision. Digesta of the ileum and cecum were collected and stored at −80 °C until analysis. Intestinal tissues were fixed in 10% phosphate-buffered formalin and stored at room temperature (Huang et al. 2014) for histological examination.

#### 2.4.2. Laying Performance

Daily feed intake, egg production, and egg weights were measured throughout the experimental period. The actual daily feed intake per group replicate was measured and used to calculate the average daily feed intake (ADFI). Laying rate is expressed as average hen-day production, calculated from the total number of eggs divided by the total number of days. Feed conversion ratio was expressed as grams of feed consumed per grams of eggs produced.

#### 2.4.3. Egg Quality

Egg quality was evaluated at the end of trial. A total of 72 eggs were randomly collected from each treatment (*n* = 72, 6 per replicate) in order to evaluate egg quality. Egg weight, Haugh unit (HU), yolk color, shell strength (measured with an eggshell strength tester), shell thickness (measured with a spiral micrometer), and shape index (determined using Vernier calipers) were evaluated. Crude protein, water, crude fat, ash, calcium, phosphorus, and the cholesterol levels of the eggs were also evaluated.

#### 2.4.4. Antioxidative Stress

Reactive oxygen species (ROS), total superoxide dismutase (T-SOD), glutathione peroxidase (GSH-PX) activity, and malondialdehyde (MDA) concentrations in either ileum or cecum were measured to estimate the oxidative status. To assess ROS, chemiluminescence (CL) derived from luminol was measured as an indicator of radical formation. Determination of T-SOD, GSH-Px, and MDA levels was accomplished using an assay kit from the Nanjing Jiancheng Bioengineering Institute. The specific procedures were followed according to the manufacturer’s instructions.

#### 2.4.5. Gut Histological Analysis

Ileum and cecum were fixed in 10% phosphate-buffered formalin, and embedded in paraffin blocks. The samples were then sectioned to a thickness of 5 mm, and stained with hematoxylin and eosin. Villus height, villus width, and crypt depth were measured on the stained sections using a light microscope fitted with an image analyzer (Image Pro Plus 6.0, Media Cybernetics, Bethesda, MD, USA).

### 2.5. Statistical Analysis

All results were statistically compared with an ANOVA test using SAS 9.4. A one-way ANOVA, followed by Duncan’s multiple comparison test, was used to evaluate different means among treatments. Differences were considered to be significant at *p* < 0.05.

### 2.6. Ethical Statement

This study is approved by the Scientific Ethic Committee of Huazhong Agricultural University (ethical approval number is HZAUCH-2017-009).

## 3. Results

### 3.1. Laying Performance

Laying performance data are summarized in Table 2. Dietary supplementation with *C. butyricum* (T1) not only reduced ADFI, but also significantly increased feed conversion (*p* = 0.0341). However, there was no apparent effect on the mortality of hens during the experimental period (*p* = 0.3445). In addition, laying rate (*p* = 0.4777), average egg weights (*p* = 0.4923), and average cracked egg percentage (*p* = 0.0957) were not influenced by dietary probiotic supplementation compared with the CON group.

### 3.2. Egg Quality

The external and internal egg qualities for layers are presented in Table 3. Compared with CON, there were no significant differences in egg quality in T2. In T1, dietary *C. butyricum* supplementation decreased eggshell strength (*p* = 0.0044) and yolk color (*p* = 0.055), and increased the CP% of albumen (DM) (*p* = 0.0809), but no statistically significant changes were noted in any of the other egg quality traits examined.

### 3.3. Antioxidative Stress

Table 4 shows the antioxidative stress status of the serum, ileum, and cecum of laying hens. The data indicate that dietary *S. boulardii* and *P. acidilactici* (T2) supplementation did not have an effect on the biomarkers of antioxidative stress. However, *C. butyricum* supplementation (T1) decreased ROS levels in both ileum (*p <* 0.01) and cecum (*p* < 0.01), as well as reduced serum MDA (*p* < 0.05). No statistically significant differences were observed for T-SOD and GSH-PX in laying hens (*p* > 0.05).

### 3.4. Histological Analysis of the Gut

**Figure 1:**
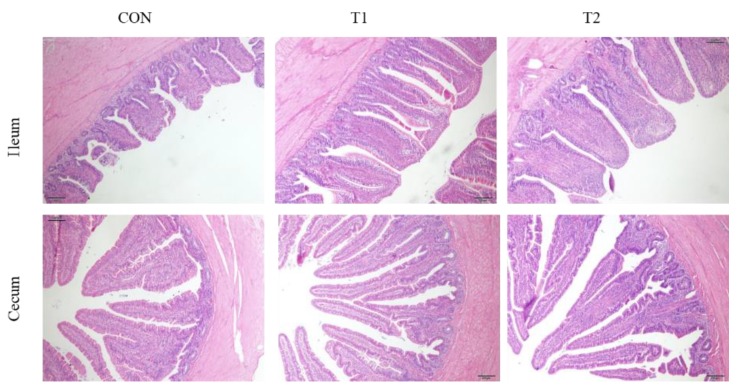
Effect of different probiotics on ileum and cecum morphology in laying hens. CON = basal diet (BD), T1 = BD plus 0.5 g/kg diet *C. butyricum* preparation, T2 = BD plus 0.1 g/kg diet *P. acidilactici* and 0.05 g/kg *S. boulardii* preparation.

As presented in Figure 1 and Table 5, dietary probiotic supplementation improved the health and microscopic structure of the ileum and cecum. The villi were observed to be short and thin in the ileum of the CON, whereas they were taller and a greater proportion were intact as observed in the ileum of groups A and B alike. Similarly, the villi in the cecum of hens from groups A and B were higher than in CON. Furthermore, villus height and villus height/crypt depth ratio (villus/crypt) were significantly increased in groups A and B compared with CON in the ileum and cecum (*p* < 0.05) (Table 5).

### 3.5. mRNA Levels of Pro-Inflammatory Cytokines in the Intestinal Tract

Gene expression levels of four major inflammatory cytokines involved in intestinal tissues were measured. Included in the assays were interleukin-6 (IL-6), interleukin-1β (IL-1β), tumor necrosis factor-α and/β (TNF-α/β). There were no statistically significant differences in gene expression observed between T1, T2, or CON.

## 4. Discussion

In recent years, the demand for eggs is no longer an issue of quantity, but has become increasingly focused on egg quality. The extensive use of antibiotics in poultry farms has not only increased the safety risks associated with egg consumption, but has also decreased egg quality. Moreover, microbial antibiotic resistance, caused by the overuse of antibiotics, is one of the biggest threats to global health, food security, and development [26]. Therefore, alternatives to antibiotics are critically needed. An important alternative to antibiotics, probiotics have been receiving more and more attention in the field of animal husbandry. Previous studies have suggested that *C. butyricum* and *S. boulardii* could improve growth performance of broiler chickens [18,27,28,29]. A few studies have also demonstrated beneficial effects of dietary *C. butyricum* supplementation on the laying performance of hens [11,30]. However, there are very limited studies examining the effects of *S. boulardii* on laying hens. Conversely, several studies have shown that the addition of butyric acid bacteria to the diet has no significant effect on improving the performance of agricultural animals [31].

In the present study, diets supplemented with *C. butyricum* or a combination of *S. boulardii* and *P. acidilactici* were examined. Diets supplemented with *C. butyricum* reduced ADFI and increased feed conversion, which is similar to the results in broilers in Mervat et al. [32]. However, the combination of probiotics had no effect on laying performance. Compared with other studies, the scale of the animal experiments performed here was lager, lending confidence in our conclusion that *C. butyricum* exerts positive effects on laying performance in hens. The significant increase in the crude protein content of albumen means the egg quality of *C. butyricum*-treated laying hen was improved.

Antioxidative stress is one of the important factors affecting the performance and egg quality of laying hens. Reactive oxygen species are widely accepted to be detrimental for health [33,34]. Studies have indicated that dietary probiotics are effective in counteracting the adverse influences of antioxidative stress [35], and promoting the activities of antioxidant enzymes [36]. Here, it was observed that *C. butyricum* significantly decreased the levels of ROS in both the ileum and cecum. Although the major antioxidant enzymes (T-SOD and GSH-PX) were apparently unaffected by probiotic supplementation, the levels of MDA, a cellular endproduct of lipid peroxidation and an important indicator of stress [37], were significantly decreased. These results may indicate that *C. butyricum* is capable of reducing oxidative stress in the intestines of the laying hens by reducing the degree of intestinal lipid oxidation as opposed to by accelerating the depletion of ROS.

The health status of the mucosa and the microscopic structure can be good indicators of the response of the intestinal tract to active substances in feed [38]. Previous studies have indicated that probiotics may be useful for improving the gut health of laying hens [35] and broiler chickens [16]. Here, it was observed that probiotic supplementation was able to significantly improve the intestinal histological morphology of laying hens (Figure 1 and Table 5). In our study, we found that dietary probiotic supplementation increased the villus height and villus height/crypt depth ratio in both ileum and cecum, which may reflect better epithelial growth after probiotic supplement [39,40].

**Figure 2:**
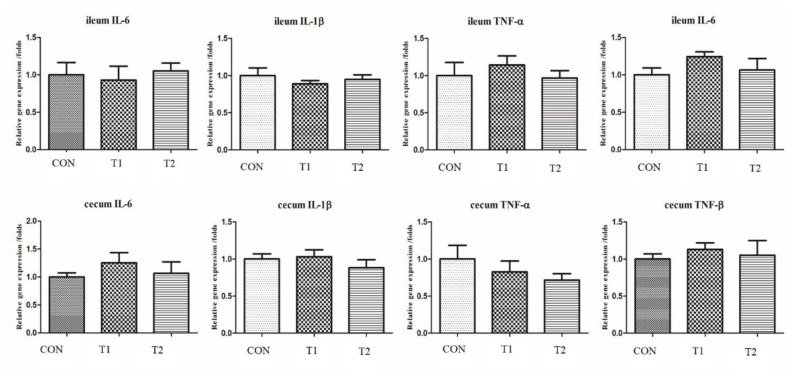
Effect of different probiotics on the mRNA levels of pro-inflammatory cytokines. CON = basal diet (BD), T1 = BD plus 0.5 g/kg diet *C. butyricum* preparation, T2 = BD plus 0.1 g/kg diet *P. acidilactici* and 0.05 g/kg *S. boulardii* preparation.

The levels of pro-inflammatory cytokines in the intestinal tract were within normal ranges (Figure 2). These results indicate that all hens were in good health, which would explain the lack of difference in the observed mortality (*p* = 0.3445, Table 2). Deng et al. (2012) demonstrated that the level of serum TNF-α in hens was reduced if probiotics were included in the diet under heat stress [35]. As this study was conducted during winter, none of the hens were under heat stress. In addition, our results also show that not all layers were experiencing oxidative stress.

## 5. Conclusions

According to our findings, we concluded that the use of probiotics containing both *C. butyricum* and a combination of *S. boulardii* and *P. acidilactici* in the laying hen diet was beneficial in enhancing the development of the intestine. In particular, dietary *C. butyricum* has significant beneficial effects on the performance of laying hens, and contributes to the improvement of normal gut morphology. However, the beneficial effects on the production performance of treatment with *S. boulardii* and *P. acidilactici* were not detected during the five-week experimental period, and this may be due to the experimental time being too short, but this result also gives us some useful guidance, as not all probiotics can be used in laying hen production, and the right choice for effective and safe probiotics is critical, and more comprehensive research needs to be performed in the future.

## Figures and Tables

**Table 1:** Composition and nutrition levels of the based diet ^1^.

Items	Content	Items	Content
Ingredients	%	Nutrient levels	
Corn	63	ME/(MJ/kg)	10.92
Soybean meal	24	Crude protein, %	15.73
Limestone	8	Lysine, %	0.82
Premix ^2^	5	Methionine, %	0.41
Total	100	Calcium, %	3.32

^1^ Values are expressed on an air-dried basis. ^2^ The premix provided the following per kg of diets: 17.0 × 10^4^ IU VA; 5.04 × 10^4^ to 10.0 × 10^4^ IU VD3; 366 mg DL-α-tocopheryl acetate; 48.0 mg menadione nicotinamide bisulfite (MNB, vitamin K3); 172 mg pantothenic; 32.1 mg thiamine nitrate; 97.2 mg vitamin B2; 425 mg nicotinamide; 144 mg Cu; 640 mg Fe; 1620 mg Mn; 1520 mg Zn.

**Table 2:** Different probiotics effect on laying performance of laying hens.

Item	CON	T1	T2	*p*-Value
ADFI	105.5 ± 1.80 ^a^	104.1 ± 1.14 ^b^	105.2 ± 1.61 ^ab^	0.083
Average egg weights (g)	57.2 ± 0.42	57.1 ± 0.46	57.3 ± 0.34	0.4923
Feed conversion (g of feed/g of egg)	1.97 ± 0.04 ^a^	1.92 ± 0.03 ^b^	1.95 ± 0.03 ^ab^	0.0341
Laying rate (%)	93.8	94.4	94.5	0.4777
Mortality (%)	2.01	1.83	2.44	0.3445
Average cracked egg percent/%	0.10	0.07	0.09	0.0957

^a,b^ Means without a common superscript with a row differ significantly (*p* < 0.05). CON = basal diet (BD), T1 = BD plus 0.5 g/kg diet *C. butyricum* preparation, T2 = BD plus 0.1 g/kg diet *P. acidilactici* and 0.05 g/kg *S. boulardii* preparation. ADFI = average daily feed intake.

**Table 3:** Effect of different probiotics on the egg quality of laying hens.

Item	CON	T1	T2	*p*-Value
Egg shape index	77.81 ± 1.15	76.44 ± 3.01	77.07 ± 0.90	0.234
Eggshell strength, kg/cm^2^	4.77 ± 0.27 ^a^	4.41 ± 0.33 ^b^	4.86 ± 0.37 ^a^	0.0044
Haugh unit	83.20 ± 5.94	82.65 ± 5.82	82.98 ± 4.09	0.9678
Albumen height, mm	7.08 ± 0.89	6.93 ± 0.78	6.95 ± 0.58	0.8791
Yolk color	7.33 ± 0.26 ^a^	7.07 ± 0.27 ^b^	7.11 ± 0.29 ^ab^	0.055
Eggshell thickness, um	346.42 ± 8.16	347.57 ± 11.66	344.68 ± 8.28	0.7569
Yolk percentage, %	25.80 ± 0.72	26.46 ± 1.53	26.14 ± 0.93	0.3553
Yolk CP%/DM	30.53 ± 0.55	31.49 ± 1.48	30.57 ± 1.48	0.3479
Albumen CP%/DM	81.06 ± 1.63 ^b^	82.65 ± 0.91 ^a^	82.27 ± 0.57 ^ab^	0.0809
Yolk Fat%/DM	55.97 ± 1.89	54.52 ± 1.77	55.77 ± 1.64	0.1091
Cholesterol content of yolk, %	3.16 ± 0.65	2.90 ± 0.17	2.94 ± 0.21	0.3046

^a,b^ Means without a common superscript with a row differ significantly (*p* < 0.05). CON = basal diet (BD), T1 = BD plus 0.5 g/kg diet *C. butyricum* preparation, T2 = BD plus 0.1 g/kg diet *P. acidilactici* and 0.05 g/kg *S. boulardii* preparation. CP = crude protein, DM = dry matter.

**Table 4:** Different probiotics effect on antioxidative status in serum, ileum, and cecum of laying hens.

	Item	CON	T1	T2	*p*
Serum	ROS (U/mL)	5.64 ± 1.25	6.60 ± 2.39	6.09 ± 3.28	0.7313
	T-SOD (U/mL)	80.37 ± 12.01	81.79 ± 8.09	82.83 ± 10.8	0.4654
	MDA (nmol/mL)	16.82 ± 4.57 ^a^	13.40 ± 3.86 ^b^	15.80 ± 5.56 ^ab^	0.0386
Ileum	ROS (U/mL)	4.12 ± 0.47 ^a^	3.57 ± 0.34 ^b^	3.94 ± 0.48 ^a^	0.0017
	T-SOD (U/mL)	101.27 ± 13.4	88.04 ± 23.69	111.56 ± 22.96	0.3091
	GSH-PX (U/mL)	132.00 ± 103.96	159.34 ± 103.71	67.75 ± 42.2	0.1613
Cecum	ROS (U/mL)	3.81 ± 0.41 ^a^	3.08 ± 0.24 ^b^	3.64 ± 0.34 ^a^	0.0009
	T-SOD (U/mL)	57.87 ± 16.16	52.09 ± 15.97	55.00 ± 14.44	0.5541
	GSH-PX (U/mL)	117.08 ± 74.91	57.74 ± 30.00	143.12 ± 160.82	0.3833

^a,b^ Means without a common superscript with a row differ significantly (*p* < 0.05). CON = basal diet (BD), T1 = BD plus 0.5 g/kg diet *C. butyricum* preparation, T2 = BD plus 0.1 g/kg diet *P. acidilactici* and 0.05 g/kg *S. boulardii* preparation. ROS = reactive oxygen species, T-SOD = total superoxide dismutase, GSH-PX = glutathione peroxidase, MDA = malondialdehyde.

**Table 5:** Effect of different probiotics on ileum and cecum morphology in laying hens.

Intestinal Segment	Item	CON	T1	T2	*p*-Value
ileum	villus height	488.51 ± 92.18 ^b^	650.60 ± 169.29 ^a^	649.47 ± 191.44 ^a^	<0.0001
	crypt depth	187.10 ± 37.92 ^b^	196.18 ± 49.84 ^ab^	207.74 ± 54.222 ^a^	0.0008
	villus width	317.38 ± 115.76	310.19 ± 77.30	354.11 ± 109.75	0.508
	villus/crypt	2.77 ± 0.78 ^b^	3.58 ± 1.33 ^a^	3.24 ± 1.01 ^a^	0.0026
cecum	villus height	1178.39 ± 187.56 ^c^	1531.50 ± 287.69 ^a^	1310.61 ± 238.16 ^b^	<0.0001
	crypt depth	197.16 ± 38.93	186.64 ± 36.45	193.73 ± 36.09	0.3794
	villus width	322.65 ± 80.66 ^a^	282.21 ± 91.54 ^b^	293.17 ± 85.75 ^ab^	0.0338
	villus/crypt	6.20 ± 1.60 ^b^	8.76 ± 2.38 ^a^	7.06 ± 2.14 ^b^	<0.0001

^a–c^ Means without a common superscript with a row differ significantly (*p* < 0.05). CON = basal diet (BD), T1 = BD plus 0.5 g/kg diet *C. butyricum* preparation, T2 = BD plus 0.1 g/kg diet *P. acidilactici* and 0.05 g/kg *S. boulardii* preparation.
